# New Innovation: Custom Titanium Zygomaticomaxillary Complex (ZMC) Plate for Facial Reconstruction

**DOI:** 10.7759/cureus.59379

**Published:** 2024-04-30

**Authors:** Sara Eliseo, Ahmed Mansour, Marshall G Miles

**Affiliations:** 1 Osteopathic Medicine, Philadelphia College of Osteopathic Medicine, Moultrie, USA; 2 Plastic & Reconstructive Surgery, Lehigh Valley Health Network, Allentown, USA

**Keywords:** complex maxillofacial fractures, zmc plate, facial reconstruction, custom titanium implant, zmc fracture

## Abstract

Zygomaticomaxillary complex (ZMC) fractures typically result from traumatic injuries, such as motor vehicle-related incidents, assaults, falls, and sports-related injuries. These fractures characteristically occur along suture lines where the zygomatic bone borders the frontal bone, maxilla, temporal bone, and sphenoid bone, resulting in a “tetrapod” fracture pattern that can be surgically fixated utilizing one, two, and three-point plate and screw fixation. However, fractures with complete loss of bone stock are less common, and standardized methods of fixation are not suitable for such complex fractures. Here, we present an interesting case of implantation of a custom-made alloplastic implant in a patient with complex ZMC fractures with loss of bone stock. A 52-year-old male sustained a traumatic gunshot wound to the face, resulting in significant destruction of bones involving the left orbital floor, left lateral orbital wall, and left zygomatic arch. Routine plating was not feasible, so a custom spanning plating system by DePuy Synthes (Synthes USA Products, LLC, West Chester, PA) was designed using the patient’s CT scans. The patient recovered well with no complications. This case illustrates the successful application of patient-specific custom plates for complex ZMC fractures when standard plating methods are not suitable.

## Introduction

Zygomaticomaxillary complex (ZMC) fractures are less common since the development of airbags but are still common facial fractures that result from traumatic injury [1]. Anatomically, they comprise fractures of the zygomatic arch, infraorbital rim, lateral orbital rim, and anterior and posterior maxillary sinus walls. Fractures of the zygomatic bone often occur at the suture lines along where it borders the frontal bone, maxilla, temporal bone, and sphenoid bone, resulting in a tetrapod fracture pattern, despite the overused misnomer “tripod fracture” [2, 3]. Since an intact zygoma is essential in maintaining orbital integrity and projection of the midface, fractures of the ZMC may inevitably result in orbital defects like enophthalmos, hypoglobus, diplopia, and restricted range of eye movement [2-4].

Traditional methods of surgical fixation of ZMC fractures comprise one, two, and three-point fixation reduction with a plate and screw [3, 5]. Standard plates are limited by their difficulty with bending to fit the contour of the patient, which can result in malreduction [6]. Additionally, while these methods are suitable for ZMC fractures that follow the typical tetrapod fracture pattern and have substantial bone stock, they are less useful in the repair of complex fractures, in which loss of quality bone stock complicates the situation dramatically.

## Case presentation

Patient presentation

**Figure 1:**
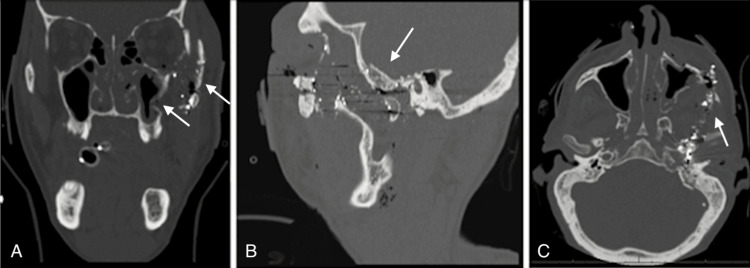
Preoperative CT images of left ZMC fractures (white arrows) in coronal (A), sagittal (B), and axial (C) views CT: computed tomography; ZMC: zygomaticomaxillary

A 52-year-old male presented to the emergency department after a gunshot wound to the left maxillofacial region with an extension to the left skull base. The patient sustained extensive injuries to his brain, including hemorrhagic parenchymal contusions in the left temporal lobe and left cerebellar hemisphere, near-complete effacement of the fourth ventricle, intraventricular hemorrhage, a thin subdural hygroma, and an acute subdural hematoma. His CT scan also revealed probable injury to the left internal auditory canal, left inner ear structures, left internal carotid artery, and left jugular foramen. The gunshot wound resulted in a minimally displaced left subcondylar fracture and complex ZMC fractures with significant loss of bone and diffuse comminution of bones involving the left orbital floor, left lateral orbital wall, and left zygomatic arch, as seen in Figure 1. The plastic surgery team was consulted for the management of this ZMC fracture, and the oral and maxillofacial surgery team was consulted to address the subcondylar fracture. However, due to the significant amount of swelling and medical instability of the patient upon initial presentation, the patient was re-evaluated for surgical intervention by both specialties after extubation, five days following admission. The patient was found to have a reproducible bite that was not clinically affected by the subcondylar fracture, and non-operative management was pursued. Regarding the ZMC fractures, a physical exam showed periorbital ecchymosis, swelling, and step-offs. Inlet and outlet wounds were centered in the left malar region with exposed bone and were closed primarily by the trauma team. The left malar region showed obvious depression. The patient possessed a left-sided facial nerve palsy from his injury, consistent with lip drooping, and an asymmetric smile. He also was unable to close the eyelid or raise the brow by command and had a restriction in lateral gaze without signs of entrapment from the orbital floor fracture. Other extraocular muscles were intact.

Sub-urgent exploration and open reduction internal fixation (ORIF) of the left ZMC with plate and screw fixation was recommended due to the substantial displacement of the fractures. However, due to the significant degree of forceful impact and subsequent loss of bone volume, routine plating was not feasible.

**Figure 2:**
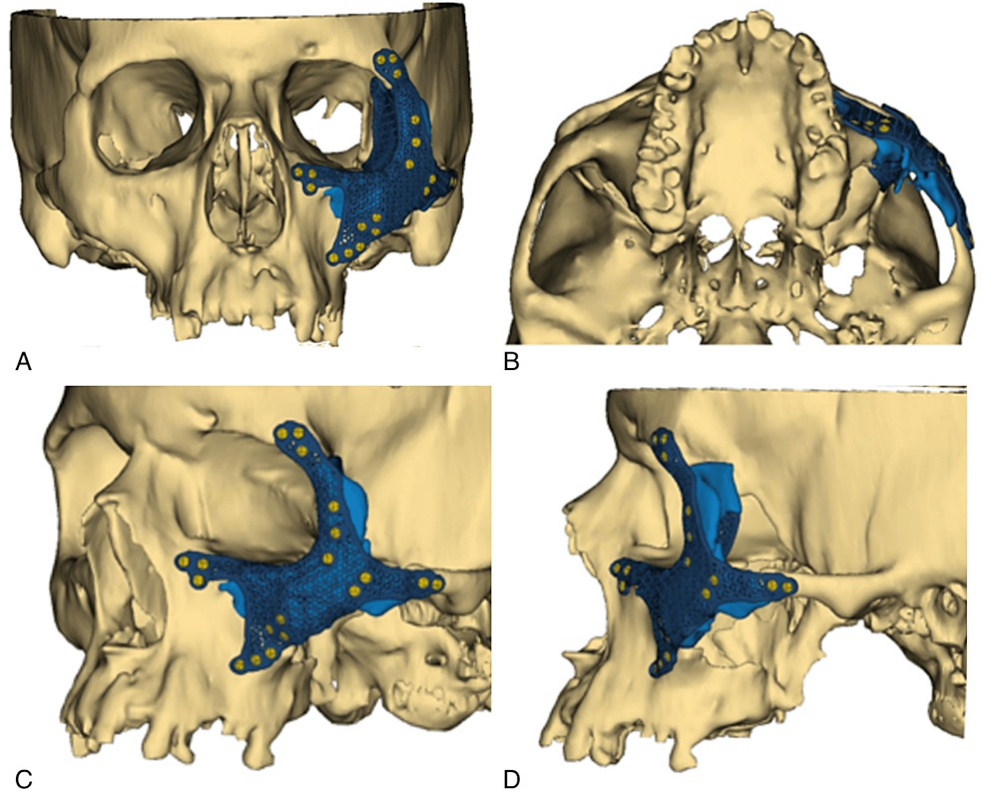
Custom spanning 3D plating design (blue) Frontal (A), inferior (B), left oblique (C), and lateral (D) views are shown above.

**Figure 3:**
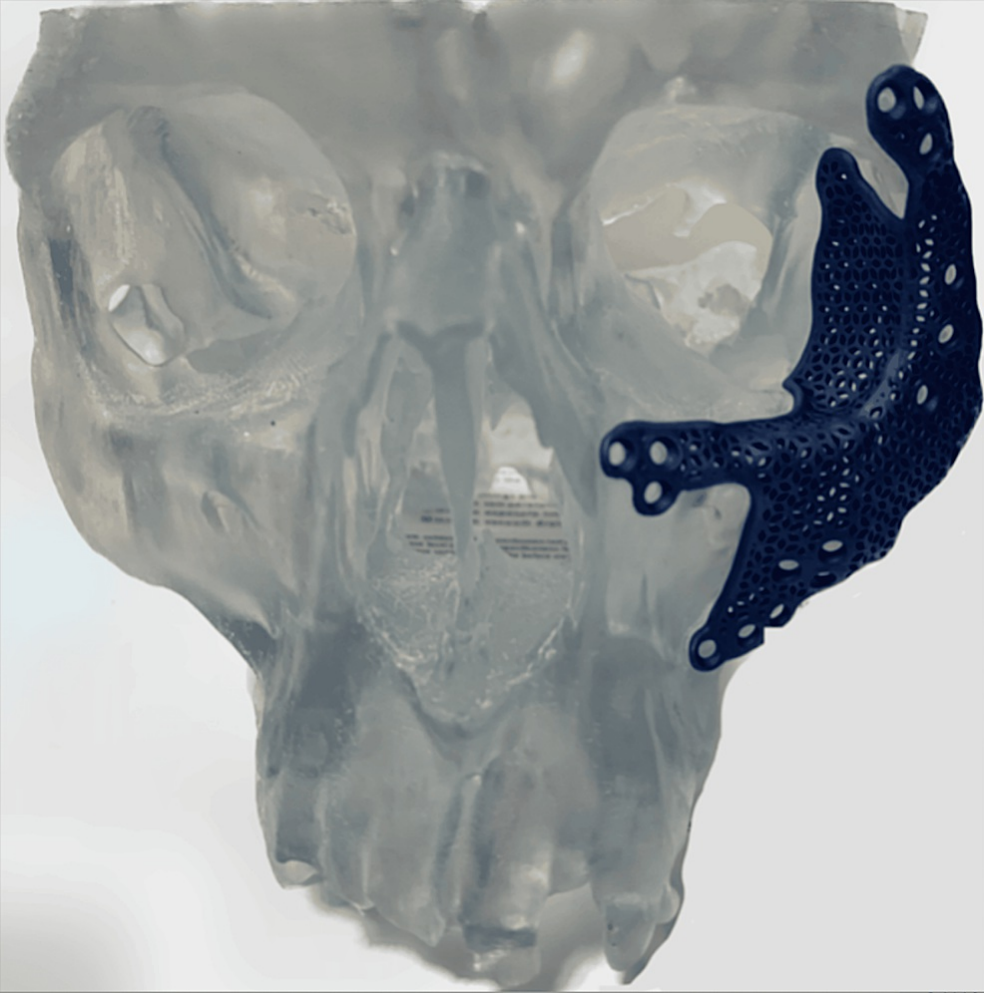
Custom spanning titanium plating system

A custom spanning plating system was designed in collaboration with DePuy Synthes (Synthes USA Products, LLC, West Chester, PA). The initial video visit consisted of a review of the patient's preoperative CT scans, and 3D reconstructed images of the facial fractures were developed. Based on the 3D renderings of the fracture pattern, a plate was designed that bridged over the heavily comminuted fracture segments onto the stable bony buttress of the zygomaticofrontal, zygomaticomaxillary, and zygomaticotemporal regions, as seen in Figure 2. We were also able to choose the location of the screw holes we deemed necessary for the plate's fixation. The custom titanium plate can be seen in Figure 3. 

Treatment

The patient underwent exploration and ORIF of the left ZMC and zygomatic arch fractures four weeks after the initial injury. Incisions were made along the previous gunshot wound scar at the left infraorbital and malar regions, the left lateral brow and the left gingivobuccal sulcus to expose the subciliary/subtarsal regions. The incisions were deepened down to the orbital rim, floor, and frontozygomatic suture line, and the periosteum was elevated from the regions. Through the same infraorbital and malar incisions, we assessed the significantly depressed left zygomatic arch fracture. Dissection proceeded over the lateral fracture of the zygomatic arch in the sub-periosteal plane, and the fracture was anatomically reduced with Gillies elevators. As anticipated, the reduction was not firm due to the significant loss and destruction of bone. Synthes screws were placed at multiple fixation sites to anchor the custom titanium plate, and rigid internal fixation of the ZMC fracture was achieved.

Upon exploration of the left orbital floor and left lateral orbital wall fractures, the orbital floor was found to be without any significant defects. However, the left lateral orbital wall fracture revealed total loss of structurally critical bone that would lead to devastating enophthalmos if not treated. As such, custom alloplastic titanium hardware and screws were used at multiple sites to internally fixate and reconstruct the orbital wall. To avoid a postoperative cicatricial ectropion, which was even more critical in this case of facial nerve injury, a left lateral canthoplasty was performed by suturing the lateral canthus to the titanium plate.

Results

**Figure 4:**
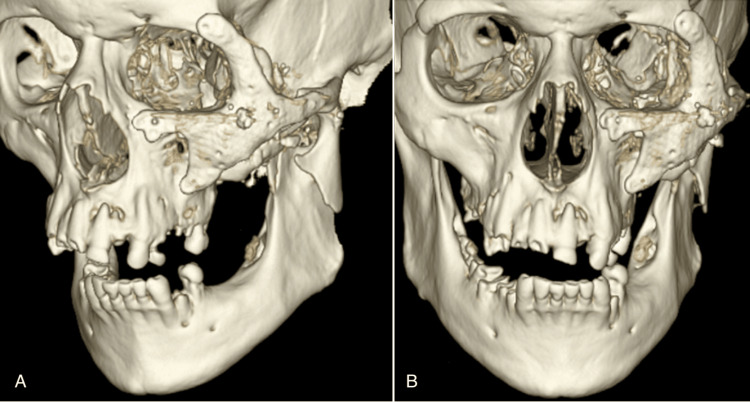
Postoperative day one 3D CT images show closed fixation with the placement of a titanium implant. Left oblique (A) and frontal (B) views are shown.

Postoperatively, the patient recovered well with no complications. Extraocular muscles remained at baseline with only limited restriction in left abduction, consistent with his preoperative evaluation; this was evaluated by the ophthalmology team and deemed to be secondary to nerve contusion and/or muscle injury from the gunshot mechanism. The 3D CT reconstruction images showed good alignment of the implant (Figure 4).

## Discussion

Reconstructive plating devices, typically made of titanium, have previously been used for more complex facial injuries. However, the large volume of titanium facial implants may cause increased stress and lead to increased bone resorption [5]. Additionally, polydioxanone (PDS) sheets and 3D-printed titanium mesh using a mirroring technique from the unaffected side have proven useful in the reconstruction of orbital fractures, but these are limited to smaller injuries that only span the orbit [6]. A custom titanium plate was made in this case, which has shown better anatomic fit and is more lightweight when compared to conventional fixation/reconstructive methods for the midface [7].

While there is a paucity of cases utilizing such a device for ZMC reconstruction, one case elucidated the use of a custom titanium implant for a highly comminuted mandibular fracture with significant bone gaps [8]. The implant used allowed for an excellent anatomic fit that recapitulated the native structure while also serving as a graft to bridge any bone gaps [8]. A retrospective study comparing midface symmetry and clinical outcomes in patients who underwent combined orbital and ZMC reconstruction with custom and standard implants found that reconstruction using patient-specific implants led to a smaller defect volume, however, without statistical significance [9]. This study also found that complications were slightly more frequent in the standard implant group as opposed to the custom implant group, likely due to smaller incisions and less intraoperative manipulation with custom implants [9]. 

One retrospective study by Ho-Kyung Lim et al. considered the long-term effectiveness and safety of patient-specific titanium implants in the reconstruction of maxillary, mandibular, and zygomatic complex defects, both congenital and due to trauma [10]. Patients were followed up between eight and 79 months postoperatively. Complications of 3D-printed implants occurred in three of 16 patients and included screw fracture, fixation failure, and postoperative dissatisfaction. Regarding the reconstruction of zygomatic complex fractures, one patient underwent implant removal due to a malunion of the plate with the zygoma. Another patient was dissatisfied due to excessive protrusion of the zygoma postoperatively. Both patients underwent a second surgery to improve implant placement, and the patients were satisfied with their appearance. [10]. The limitation in comparing the patients in this study to the ones described in our case is the extent of the defect. While one patient underwent ZMC reconstruction due to a traumatic injury, the fractures were not quite as extensive as the patient in our case, as the blast injury from the gunshot wound resulted in severely comminuted fractures. 

Titanium has traditionally been used for bone repair due to its high tensile and compressive strengths and similar modulus of elasticity to bone [10, 11]. Titanium 3D-printed implants by DePuy Synthes are designed with variable thickness and combined porous and continuous structures to align with the complex anatomical shape of the midface. Additionally, the TRUMATCH® orthognathic system addresses vertical maxillary positional challenges that are often seen with complex asymmetric ZMC cases, reducing the need for splints and plate bending that would otherwise add bulk to the device [12]. While this case posed an increased time expenditure in preoperative planning, the custom plate allowed for appropriate reconstruction in an instance when routine plating would not have been achievable.

## Conclusions

The patient in this case sustained complex ZMC fractures that would not have been feasible with traditional plate and screw reduction. Creating a custom titanium plate to repair the ZMC in this patient was an ideal option as it reduced the number of incisions that otherwise would have been needed to accommodate numerous plates and screws, and it allowed for a better anatomical fit and less bulky repair of the area. This case serves to illustrate the application of patient-specific custom plates for the reduction and restoration of complex ZMC fractures when traditional reductions are not practical. The use of a custom titanium plate made by DePuy Synthes was shown to be effective in the internal fixation and reconstruction of our patient’s extensive facial fractures.
